# Genome-wide identification and comprehensive analysis of the NAC transcription factor family in sunflower during salt and drought stress

**DOI:** 10.1038/s41598-021-98107-4

**Published:** 2021-10-06

**Authors:** Wenhui Li, Youling Zeng, Fangliu Yin, Ran Wei, Xiaofei Mao

**Affiliations:** Present Address: Xinjiang Key Laboratory of Biological Resources and Genetic Engineering, College of Life Science and Technology, Xinjiang University, Urumqi, 830046 China

**Keywords:** Sequence annotation, Abiotic

## Abstract

The NAC (NAM, ATAF1/2, and CUC2), is a large family of plant-specific transcription factors (TFs) that exert crucial regulatory roles in various physiological processes and abiotic stresses. There is scanty information on the role of the NAC family in sunflower (*Helianthus annuus* L.). In this study, we conducted a genome-wide survey and expression analysis of the NAC family in sunflower. A total of 150 *HaNACs* were identified in sunflower. Phylogenetic analysis to compare HaNACs with *Arabidopsis* NACs generated 15 clusters. Among them, eight membrane-bound NAC TFs with transmembrane helixes were found (designated as NTLs), which were suggested to be localized in the membrane and transferred to the nucleus through proteolysis. Notably, 12 HaNACs were potentially regulated via miR164 cleavage or translational inhibition. By analyzing RNA-seq data from Sequence Read Archive (SRA), the expression of *HaNACs* showed tissue specificity and strong response to drought stress. Additionally, phylogenetic analysis of 150 HaNACs with the previously reported NACs related to abiotic stress revealed that 75% of the abiotic stress-related NACs were clustered into the SNAC (abiotic stress-related NAC) group, and only 25% were in the Non-SNAC group. qRT-PCR further demonstrated that about 75% of the *HaNACs* in the SNAC subgroup were induced by salt and drought stress, and the expression of some *HaNACs* showed tissue specificity. These findings provide valuable information that can deepen the understanding of how NAC TFs in sunflower respond to abiotic stress.

## Introduction

Environmental stresses are the main limiting factors for the growth, productivity, and distribution of plants. One-third of the Earth's surface is classified as either arid or semi-arid, and nearly 40% of the world's land is characterized by potentially high salinity levels^1^. For sunflower, poor weather and soil conditions are among the major obstacles to high yield production^2^, and soil salinity is particularly the major constraint^3^. High salt concentration can lower the seed germination rate, limit the water absorption of plant roots, delay plant growth, and cause nutritional imbalance^4^. In addition, secondary pressures, such as oxidative damage, can exacerbate the influence of the above outcomes^5^. Drought stress is the most prevalent environmental factor limiting crop productivity^6^, and global climate change is increasing the frequency of severe drought conditions^7^. Drought stress causes the transpiration rate to exceed the water uptake, and the growth of plants will encounter water shortage. Water deficiency can cause the changes of solute concentration in cells, which leads to the disruption of water potential gradients, changes of cell volume, denaturation of protein, and disruption of membrane integrity, and eventually the plants are damaged^6^.

Globally, sunflower is the fourth most important oil crop after palm, soybean, and rapeseed, and second in Europe. A growing trend for sunflower cooking oil is attributed to the rise in sunflower production, worldwide^8^. Sunflower is considered a moderately salt-tolerant crop and is widely cultivated in saline-alkali areas around the world^9^. Reconstruction of crops can enhance their resistance to abiotic stress. However, it is still challenging to grow plants in extreme environments. A few crops can grow in highly salinized soils. There is enormous potential to develop saline-alkali characteristic crops for sunflowers based on its well-known moderate salt tolerance, compared with non-resistant crops. Limited rainfall or shortage of water for irrigation during the growing season constraints its seed yield with significant reductions^10^. Therefore, the breeding of drought tolerant cultivars will contribute to more stable sunflower production. Several studies have established sunflower regeneration and genetic modification methods, which makes it possible to use genetic engineering to cultivate salt and drought-tolerant sunflower^11–14^. However, there is also a need to find genes to endow the salt and drought resistance of plants.

Abiotic stress resistance requires the production of important functional proteins, such as osmoprotectants and regulatory proteins, that play roles in signal transduction pathways, including kinases and transcription factors (TFs)^15^. NAC **(**NAM, ATAF1/2, and CUC2) family is a plant-specific transcription factor firstly discovered in *Petunia hybrida*^16^, and involved in plant salt and drought stress response (multiple abiotic-stress responses)^17–19^. They possess a conserved NAC domain (about 150 amino acids in the N-terminal region), which usually exists in five subdomains (A, B, C, D and E). The NAC domain is responsible for DNA binding and dimer formation, whereas different C-terminal regions participate in transcriptional regulation^19,20^.

NACs have been identified from a variety of plants, such as banana^21^, *Fragaria vesca*^22^, *Fragaria* × *ananassa*^23^, *Oxytropis ochrocephala*^24^, *Sesamum indicum*^25^, *Capsicum annuum*^26^. More than 100 genes from this family have been identified in *Arabidopsis*, rice and soybean^20,27,28^. Data on sunflower genome sequences were published and released in 2017^29^, which was a breakthrough in the molecular biological research process of sunflowers. Since then, a few studies on NACs in *Helianthus annuus* are ongoing. Of note, only a study by Yuce et al.^30^ has so far reported that sunflower NAC TFs responded to chromium stress. Elsewhere, Moschen et al. found some NACs were associated with leaf senescence^31^, and a sunflower NAC was induced under drought stress^32^. However, the functions of most sunflower NAC members are unknown. This study gap prompted us to comprehensively explore the sunflower NAC family by identification, protein property, phylogenetic analysis, transmembrane helix, miRNA164 target site, tissue distribution, and analysis of abiotic stress expression profiles. This work will lay a foundation for further study on the function and regulation mechanism of NAC TFs in sunflower.

## Results

### Identification and annotation of NAC members

Initially, a total of 157 non-redundant putative NACs were identified via HMM (Hidden Markov Model) search. First, through the identification of CCD and pfam, one protein was deleted, because it lacked the NAC domain. Then, six proteins among them with incomplete NAC subdomains were also removed, after which we obtained a final total of 150 NACs with intact NAC domains from the sunflower. The number was slightly higher than that of plants such as tomato (104 NACs)^33^, *Arabidopsis* (106)^34^, sesame (87)^25^, and soybean (101)^35^. It was speculated that the presence of more NACs in the sunflower highly reflects their participation in the complex transcriptional regulation of sunflower. This phenomenon also appears to be caused by multiple gene duplication events.

Moreover, the characteristics of NAC protein sequences greatly varied in sunflower. The sequence length of 150 HaNACs ranged from 139 to 636 aa, the molecular weight from 16.16 to 72.13 kDa and the isoelectric point from 4.17 to 10.44. Detailed information on these data was shown in Table S1.

**Figure 1:**
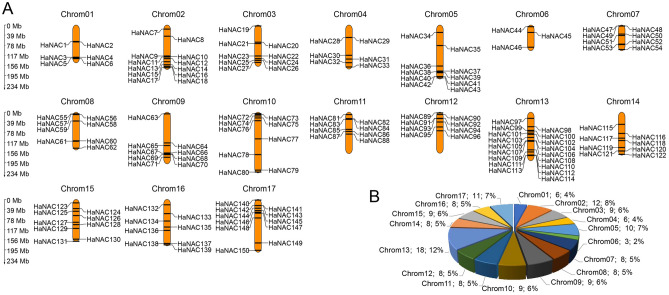
Distribution of *HaNACs* on all 17 sunflower chromosomes. (**A**) The size of each chromosome and its corresponding *HaNACs* distribution; (**B**) The pie chart showed the number and percentage of *HaNACs* on each chromosome.

Due to the lack of comprehensive standard annotations, we mapped the 150 *HaNACs* to all 17 chromosomes and named them from *HaNAC1*-*HaNAC150* based on their position on the chromosome. Among them, chromosome 13 had a maximum of 18 *HaNACs* (~ 12%), whereas chromosome 6 had only 3 *HaNACs* (~ 2%) (Fig. 1). The uneven distribution of *HaNACs* on the chromosomes reflected the diversity and complexity of the *HaNAC* gene family.

### Phylogenetic analysis of sunflower NACs

**Figure 2:**
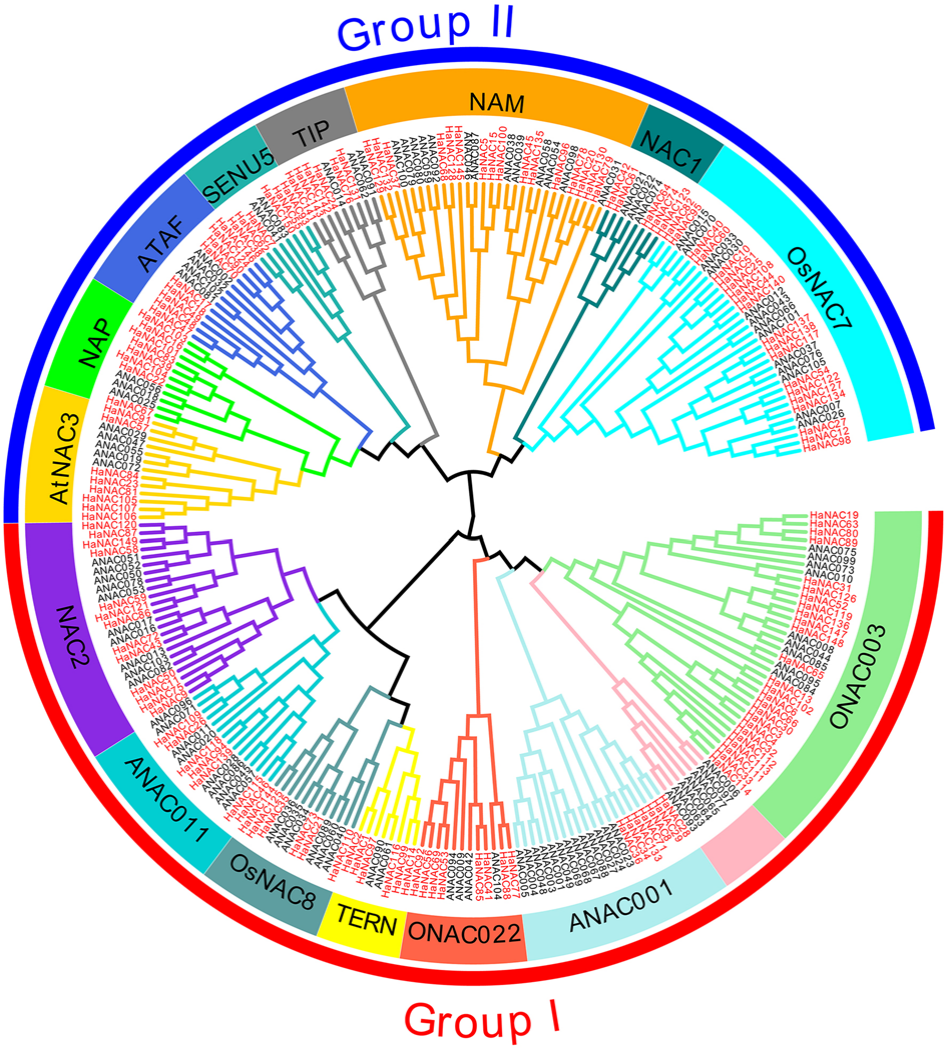
A phylogenetic tree of sunflower and *Arabidopsis* NAC proteins. The amino acid sequences of NAC proteins were aligned using the ClustalW of MEGA X, and a phylogenetic tree was generated using the neighbor-joining method with 1000 bootstrap replicates in MEGA X. Red and black fonts denoted sunflower and *Arabidopsis* NACs, respectively. All NACs were classified into two groups: Group I (red) and Group II (blue), and 15 subgroups (different colors for each clade).

In 2003, Ooka et al. established a classification system for the NAC family^20^, which has so far been applied in various plants, such as sorghum^36^, pepper^26^, and rice^37^, among others. Herein, to reveal the phylogenetic relationship and potential functional characteristics of the HaNAC family members, a phylogenetic tree was constructed using 255 full-length protein sequences from sunflower and *Arabidopsis* using MEGA X, Neighbor-joining (NJ) and Maximum Likelihood (ML). Since the trees produced by the two methods were largely similar (data not shown), only the result generated via the NJ method was shown in Fig. 2. We divided the 150 HaNACs into two groups and 15 subgroups. The largest clade was the ONAC003 subgroup containing 25 HaNACs, while the NAC1 subgroup constituted the smallest clade with 5 HaNACs and this clade only contained NACs from *Arabidopsis thaliana*. Remarkably, *Arabidopsis* NACs with the same function were clustered to the same subgroup. For instance, the AtNAC3, NAP, and ATAF subgroups contained several well-known stress-responsive NACs, including ANAC019, ANAC056 (AtNAC2), ANAC055 (AtNAC3), ANAC002 (ATAF1), ANAC081 (ATAF2), and ANAC072 (RD26). In addition, we examined other evidence to support the reliability of subgroup classification, such as gene structure and motifs, as described below.

### Gene structure and conserved motifs of sunflower NACs

**Figure 3:**
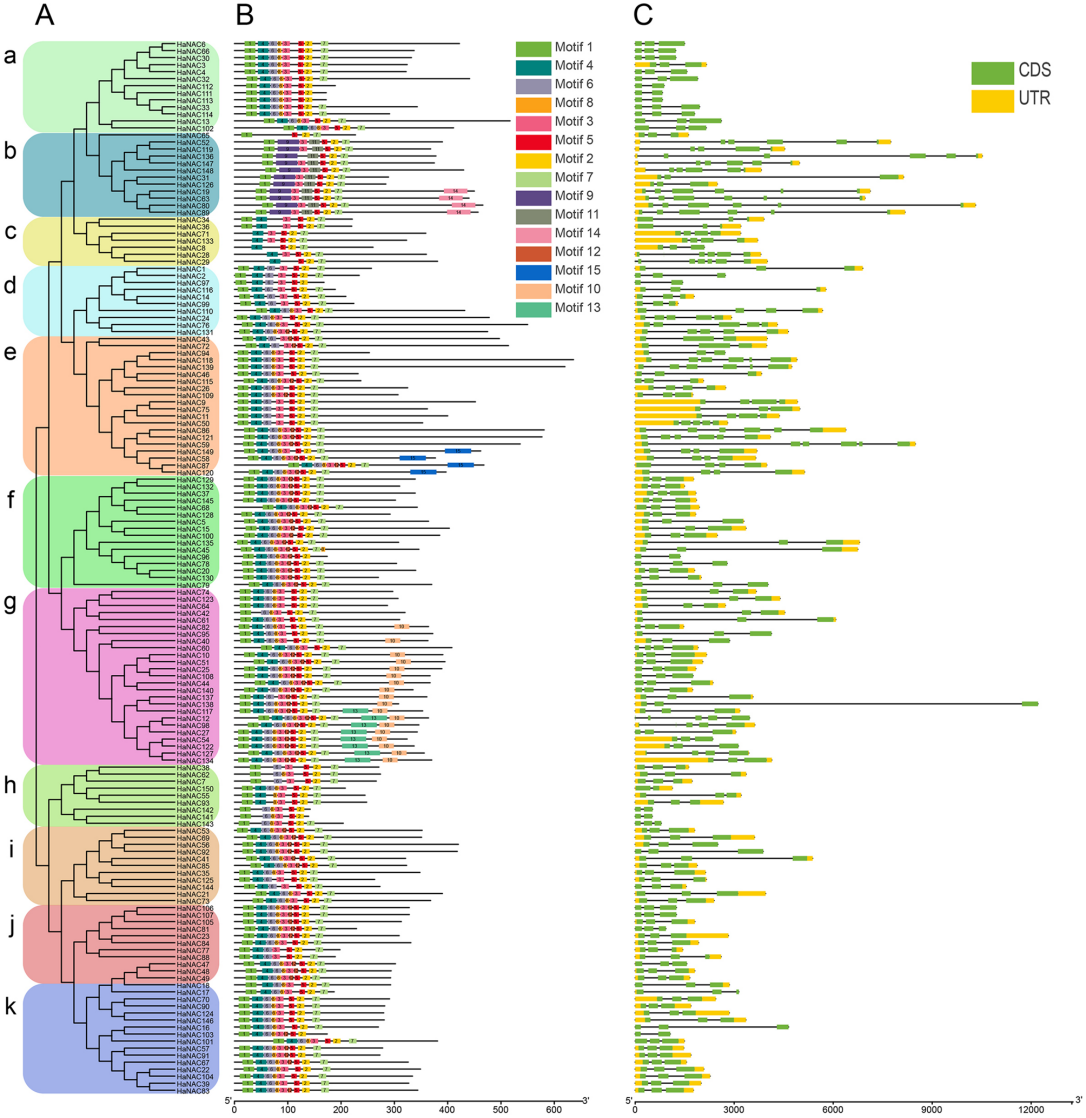
Phylogenetic tree, motif, and genetic structure of 150 HaNAC TFs. (**A**) The phylogenetic tree was constructed using the Neighbor-Joining (NJ) method and 1000 bootstrap tests. (**B**) Showing the positions of 15 motifs in the proteins. Different colors indicated different motifs. (**C**) Gene structures, exons and untranslated regions (UTR) were indicated by green and yellow boxes, and black lines indicated introns. The ruler at the bottom was used to estimate their length.

Multiple sequence alignment of the sunflower NACs demonstrated that the N-terminus of all HaNACs have a conserved NAC domain, and five subdomains (Fig. S1). To better understand the relationship between the structure and function of these HaNAC proteins, the gene structure, and conserved motifs were analyzed. The exon numbers of sunflower *NAC* genes ranged from 2 to 7, among which, most genes had three exons (Table S2). Generally, members from the same subgroup were characterized by similar exon/intron structure and gene length. For example, subgroups i and j contained three exons, whereas subgroups a, h and k contained 2 or 3 exons (Fig. 3C).

A total of 15 conservative motifs of HaNAC proteins in sunflower were shown in Fig. S2. As expected, the most closely related members of the same subfamily shared a common motif composition, which also meant that they had similar functions (Fig. 3B). Most of the predicted motifs were at the N-terminus, which was more conservative. Motif5 and motif2 were present in most HaNACs, and only specific proteins in the subgroup c did not have motif1. A phylogenetic tree of 150 sunflower NACs was constructed using the above method (Fig. 3A). It was worth noting that several special motifs (10, 13, 14, and 15) at the C-terminus were clustered together. These results not only proved the reliability of the classification, but also revealed a correlation between the subfamily and motif.

### Syntenic and evolutionary analysis of *HaNAC* gene family

**Figure 4:**
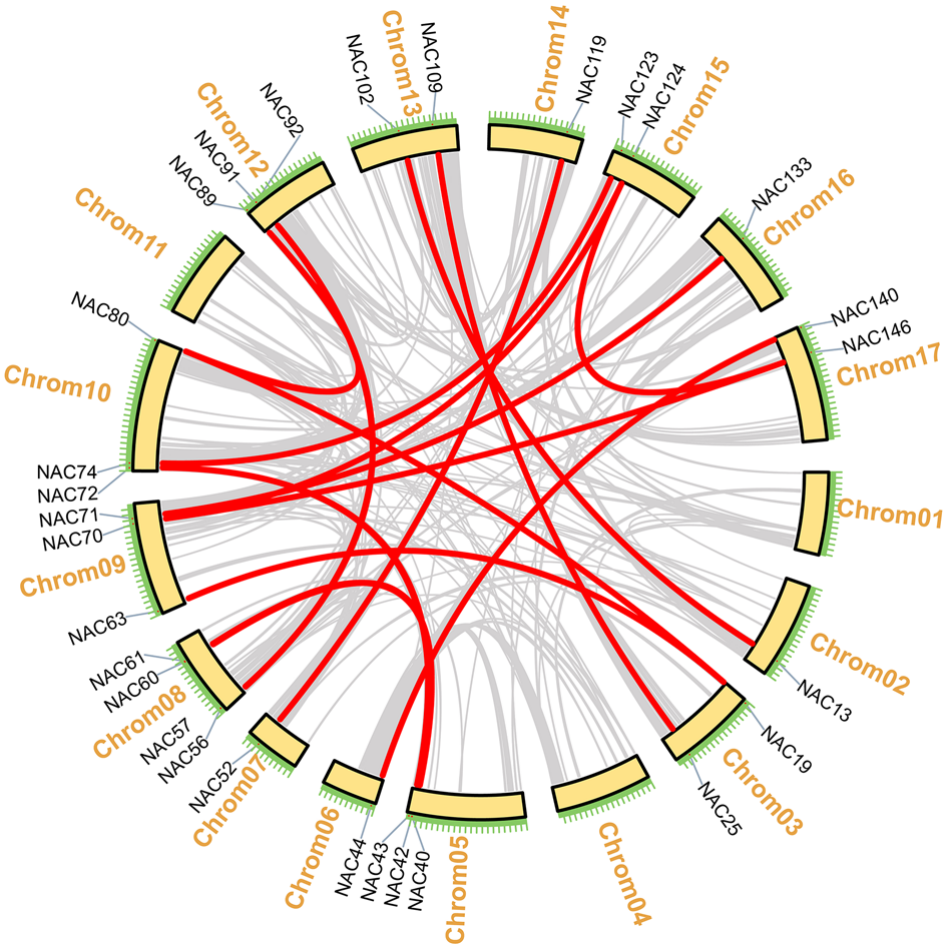
Syntenic analysis of the *HaNAC*s. The yellow box represented the chromosome, the line indicated the position of the syntenic gene pair on the chromosome, the red line denoted the *HaNAC* gene pairs, while the gray line showed other syntenic gene pairs in the sunflower.

Duplication events are related to plant evolution patterns, while tandem duplication and segmental duplication are sources of gene family expansion and genomic complexity. The duplication events of *HaNACs* were identified using blastp and MCscan. Segment duplication analysis revealed segment duplications in 17 *HaNAC* gene pairs such as *HaNAC19*–*HaNAC63*, and *HaNAC40*–*HaNAC60* (Fig. 4 and Table S3). Tandem duplication (when two genes were located in a 100 kb region on the same chromosome and separated by five or fewer genes), was found in 10 *HaNAC* gene pairs, such as in *HaNAC3*–*HaNAC4*, and *HaNAC28*–*HaNAC29* (Table S3). To evaluate the selection limits for duplicated *HaNAC* genes, we estimated ratios of the number of non-synonymous substitutions per non-synonymous site (Ka) to the number of synonymous substitutions per synonymous site (Ks). Of the 27 duplication gene pairs, 26 pairs evolved under purified selection (Ka/Ks < 1), whereas one pair evolved under positive selection (*HaNAC112*-*HaNAC113*, Ka/Ks = 1.15). The divergence time of the *HaNAC* genes indicated that the duplication event could be potentially traced back to 83.51 million years ago (Mya) and continued to 0.46 Mya. (Table S3).

### Membrane-bound HaNACs and miR164s target site prediction

Subcellular localization prediction indicated that most of the 150 NAC proteins were localized in the nucleus (Table S1), which was consistent with the characteristics of NAC as a transcription factor. Numerous MTFs (membrane-bound transcription factors) had been identified in the NAC family and were named NTL (NAC with transmembrane motif1-like)^38^. They were, in most cases bound to the membrane, and possibly released from the membrane into the nucleus by proteolysis^39^. Using the TMHMM2.0, eight NAC proteins containing a transmembrane helix were predicted, which were designated as HaNTL1-HaNTL8 (Table 1 and Fig. S3). A phylogenetic tree of NTLs from sunflower, *Arabidopsis,* and rice was generated, from which sunflower NTLs were closely related to *Arabidopsis* NTLs, indicating a similar function of these genes in the two species (Fig. S4).

**Table 1:** Putative membrane-bound NAC transcription factors in sunflower.

Name	Gene symbol	Transcript ID	Size (aa)	Transmembrane regions
HaNTL1	HaNAC24	OTG32123	478	451–470
HaNTL2	HaNAC43	OTG26785	497	466–488
HaNTL3	HaNAC59	OTG17990	536	508–530
HaNTL4	HaNAC72	OTG09643	514	484–506
HaNTL5	HaNAC76	OTG10114	550	526–548
HaNTL6	HaNAC86	OTG07933	581	553–575
HaNTL7	HaNAC101	OTG01218	381	30–52
HaNTL8	HaNAC110	OTG03048	432	382–404

**Figure 5:**
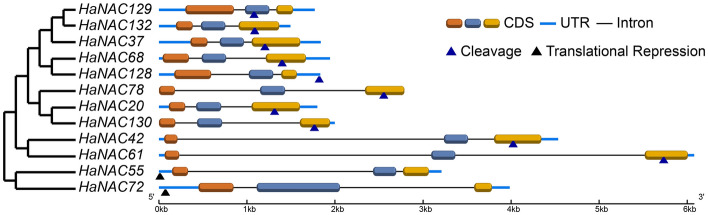
Phylogenetic tree and regulatory sites of *HaNACs* regulated by miRNA164s; Blue triangles indicated regulation of gene expression by cleavage, Black represented inhibition of mRNA translation.

Based on the current understanding, microRNAs (small non-coding RNAs of about 22 nucleotides in length) can regulate target gene expression^40^. Researchers have revealed that NAC family genes are regulated by miRNA164s^41–43^. For instance, a study by Badouin et al. in 2017 sequenced small RNAs in the sunflower genome and found four exceptionally reliable miRNA164s^29^. The regulatory relationship between these miRNA164s and *HaNACs* was predicted using the psRNATarget online tool^44^, which was a total of 12 *HaNACs* were probably regulated by these miRNA164s. Among them, ten could be regulated at the transcriptional level by mRNA cleavage, whereas the other two could be regulated at the translational level via inhibition of mRNA translation. Notably, these target sites were distributed in the 5′ untranslated regions, 3′ untranslated regions, different coding sequences, but not in the conserved NAC domains (Fig. 5). This signified the complexity in the regulation of *HaNACs* by miRNA164s.

### *Cis*-element analysis

To explore the potential functions of *HaNACs*, 24,425 *cis*-elements were detected in the promoter region (2000 bp upstream of the start codon of the gene) using PlantCare. Excluding conventional *cis*-elements (such as TATA-box and CAAT-box), the *cis*-elements with functional annotation were divided into 20 groups, including light-, methyl jasmonate-, and anaerobic-responsive elements, among others. (Tables S4 and S5). Among them, light-related *cis*-elements accounted for the largest portion (45.97%), followed by methyl jasmonate (12.11%), anaerobic (10.91%), abscisic acid (9.93%), and low temperature (3.11%). Since previous studies revealed that both methyl jasmonate and abscisic acid can play various roles in plant stress and defense^45–47^, it can be speculated that HaNACs were closely related to the photoperiod, stress, and defense of sunflower. In addition, there were some *cis*-element groups related to special tissue, such as these groups of meristem and endosperm expression, and differentiation of the palisade mesophyll cells (Table S5). Additional information on *cis*-elements was provided in Fig. S5, and Table S4.

### *HaNAC* gene expression profiles in different organs and under PEG stress

**Figure 6:**
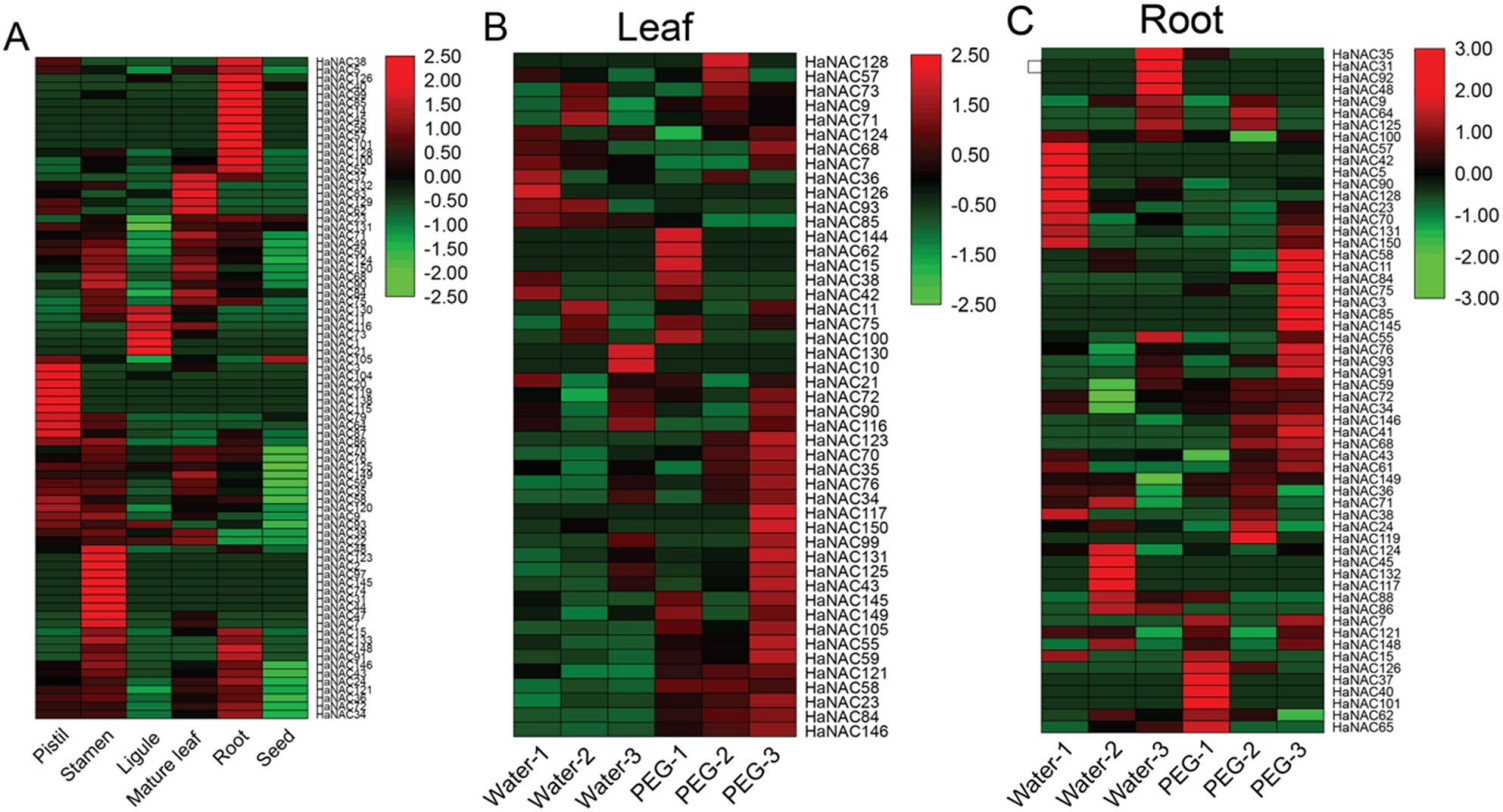
The expression profiles of *HaNAC* genes in different organs and under PEG stress based on transcriptome data from SRA. The legend represented a logarithmic normalized TPM. (**A**) *HaNAC* gene expression profiles in different organs. (**B**, **C**) *HaNAC* gene expression profiles in leaves and roots after 15% PEG stress treatment, respectively.

To infer the role of *HaNACs* in different tissues, the RNA-seq data of sunflower from the Sequence Read Archive (SRA) database were searched, downloaded, and analyzed. In total, 80 genes were expressed in the pistil, stamen, ligule, mature leaves, roots, and seeds. *HaNAC20*, *HaNAC115*, *HaNAC119,* and *HaNAC138* genes were uniquely expressed in pistil; *HaNAC2*, *HaNAC31*, *HaNAC44*, *HaNAC74*, *HaNAC97*, *HaNAC123,* and *HaNAC145* were only expressed in stamen; *HaNAC1*, *HaNAC21* in ligule; *HaNAC14*, *HaNAC45*, *HaNAC56*, *HaNAC57*, *HaNAC85,* and *HaNAC101* were only expressed in the root. Higher expressions of *HaNAC83*, *HaNAC129,* and *HaNAC132* were observed in mature leaf, but could also be found in other organs. Except for *HaNAC23* and *HaNAC105*, all genes were lowly expressed in seeds. This result could be attributed to the dormant state of seeds and low gene expression (Fig. 6A). Overall, these findings implied that different *HaNACs* might play different roles in various tissues.

To assess the function of the *HaNAC* gene under drought stress, RNA-seq data of leaves and roots treated under 15% PEG stress for 24 h with sunflower seedlings at four-leaf stage from the SRA database showed that 67 *HaNAC* genes were present in all samples, among them, 47 *HaNAC* genes were detected in leaves and 58 in roots, respectively (Fig. 6B,C). Using *p*-value < 0.05 as the standard for differentially expressed genes (DEGs), the DEGs analysis revealed 10 differentially expressed *HaNAC* genes in the leaves under PEG stress. *HaNAC58*, *HaNAC84*, *HaNAC149*, *HaNAC105*, *HaNAC57*, *HaNAC146*, *HaNAC23*, *HaNAC121*, and *HaNAC145* were up-regulated, whereas only *HaNAC85* was down-regulated (Table 2). Besides, three DEGs (*HaNAC76*, *HaNAC62*, *HaNAC24*) were up-regulated in the roots (Table 2). Of the differentially expressed *HaNAC* genes, most were up-regulated, demonstrating that these *HaNAC* genes might exert a positive regulatory role in drought response.

**Table 2:** *P*-value and fold change of differentially expressed *HaNAC* genes in sunflower after 15% PEG stress treatment with the data from SRA.

Organ	Gene	*p*-value	Fold change
Leaves	*HaNAC58*	0.0020842	2.0837142
Leaves	*HaNAC84*	0.0044662	3.7878544
Leaves	*HaNAC149*	0.0075976	1.3874092
Leaves	*HaNAC105*	0.0079363	6.0732695
Leaves	*HaNAC57*	0.0130001	3.4120673
Leaves	*HaNAC146*	0.0131316	1.9069783
Leaves	*HaNAC23*	0.0222085	2.8785682
Leaves	*HaNAC85*	0.0253651	0.6668983
Leaves	*HaNAC121*	0.0425178	1.5225648
Leaves	*HaNAC145*	0.0468288	2.0679343
Roots	*HaNAC76*	0.0011284	3.4415853
Roots	*HaNAC62*	0.0108968	1.6200786
Roots	*HaNAC24*	0.0407995	1.2328135

### *HaNACs* related to abiotic stress and their stress response

**Figure 7:**
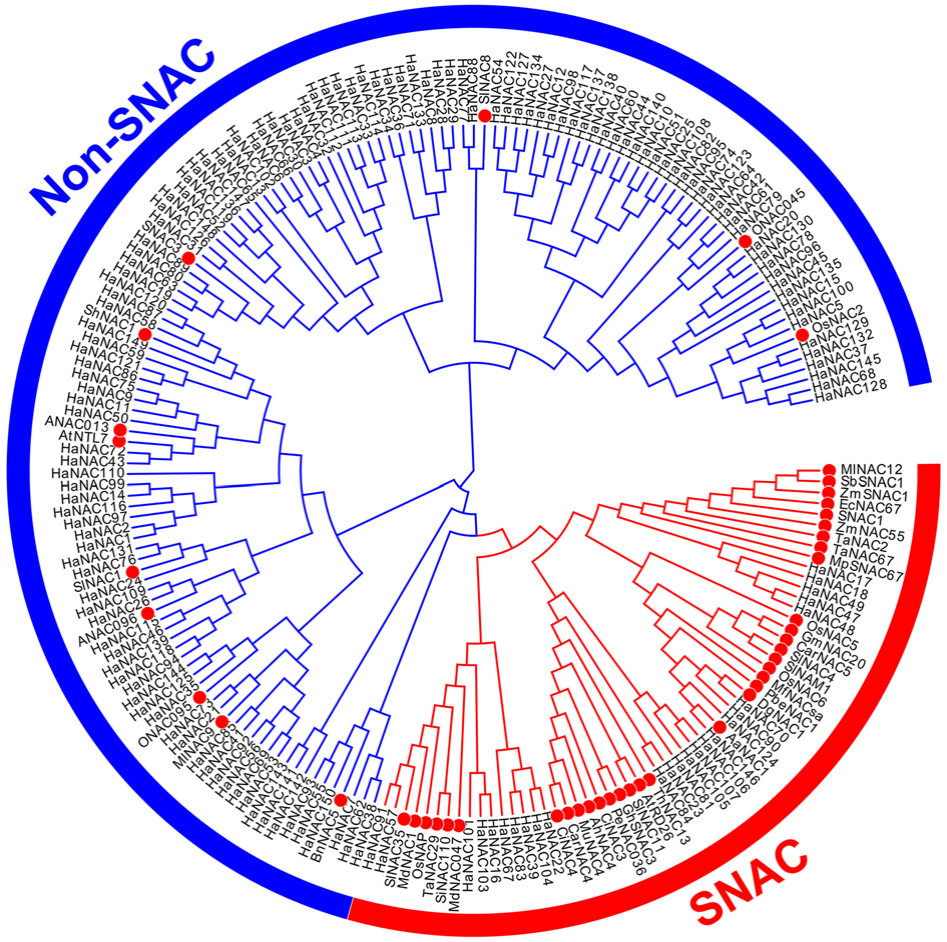
A phylogenetic tree generated using 47 reported NACs enhancing plant tolerance to abiotic stress and HaNACs from sunflower. Red dots indicated NACs reported could improve abiotic stress tolerance in plants. Most of these 47 *NAC* genes were densely distributed in the red subgroup, here called the SNAC group, and the other as Non-SNAC group.

Here, we determined which *HaNACs* potentially played a role in abiotic stress. Of note, 46 scientific papers from 2004 to 2019 have been reported that NACs could enhance plant abiotic stress resistance (mainly salt and drought resistance) (Table S6); Thus, 47 related NAC protein sequences were downloaded and a phylogenetic tree was constructed using HaNACs and these 47 NACs protein sequences. Notably, most NAC proteins enhancing abiotic resistance in plants were classified into the SNAC branch (Fig. 7). These NAC members in this branch were called abiotic stress-related NACs (SNAC). The 35 tolerant NACs were distributed in the SNAC group, while only 12 tolerant NACs were in the larger Non-SNAC group.

**Figure 8:**
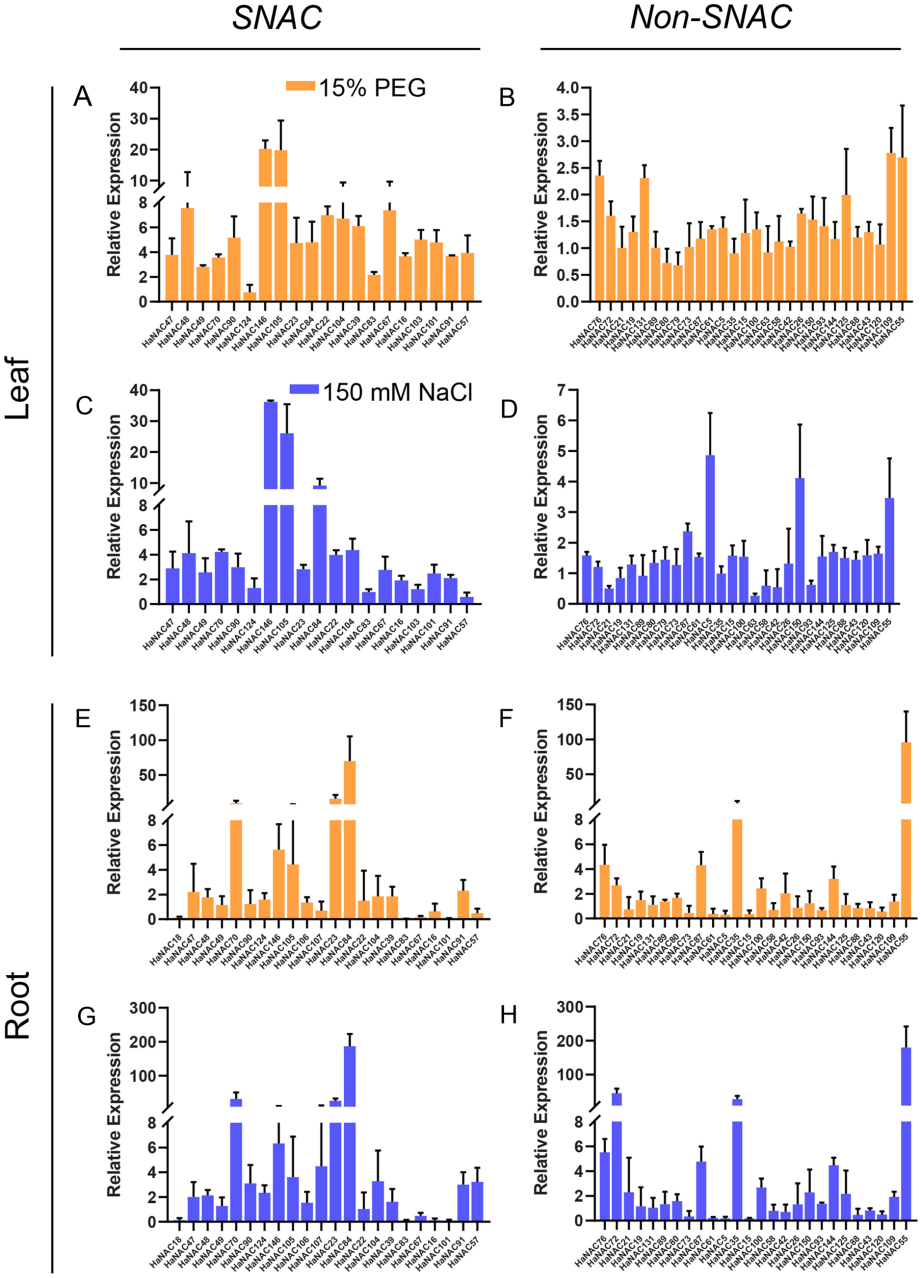
Relative expression of some *HaNAC*s under 15% PEG or 150 mM NaCl stress for 24 h, including those in the SNAC group and those in the same branch as the reported NACs in the Non-SNAC group. (**A**–**D**) showed the conditions of gene expression in leaves; (**E**–**G**) represented those in roots; (**A**, **C**, **E**, **G**) showed the results of *SNAC* gene expression; (**B**, **D**, **F**, **H**) indicated those of *Non*-*SNAC* genes; (**A**, **B**, **E**, **F**) showed the results of gene expression to PEG stress and (**C**, **D**, **G**, **H**) as the results to NaCl stress.

Based on the correlation of function with evolution, it can be assumed that the HaNACs on the SNAC branch are strongly associated with abiotic stress. Therefore, it is necessary to detect the expression of some *HaNACs* under salt- and PEG-abiotic stress, and these *HaNACs* were chosen from the SNAC group and the same branch as tolerant NACs in the Non-SNAC group. Four-leaf stage sunflowers were treated with 15% PEG or 150 mM NaCl for 24 h, and the relative expressions of these *HaNACs* were determined by qRT-PCR. Results showed that the gene expression pattern was similar under PEG and NaCl stress in the same tissues and gene expression varied widely in different tissues. In both the SNAC and Non-SNAC groups, the expressions of some genes were up-regulated after stress. Of note, the numbers and folds of up-regulated genes in the SNAC group were more and higher. In leaves, *HaNAC146* and *HaNAC105* were most significantly up-regulated, while *HaNAC70*, *HaNAC23*, *HaNAC84*, *HaNAC55*, and *HaNAC35* were most significantly up-regulated in roots (Fig. 8). These up-regulated genes might play an important role in plant response to abiotic stress.

## Discussion

Sunflower is an economically significant crop with medium salt and drought resistance^9^. Since a substantial fraction of land in the world is highly saline, improving the ability of sunflower to tolerate abiotic stress would facilitate the utility of this saline-alkali wasteland for agricultural production. The *NAC* gene family, as a plant-specific transcription factor family, plays important functions in response to abiotic and biotic stresses^39,48,49^. Limited information has been published on the functional association of the *NAC* gene family in sunflower. Herein, we reported the first study to systematically analyze the *NAC* gene family and conduct an expression profiling in sunflower using bioinformatics tools and real-time PCR technology. According to published genomic data, 150 *NAC* genes have been identified in the sunflower genome (Fig. 1 and Table S1); however, some *HaNAC* genes are yet to be found because the genome only accounts for about 80% of the entire sunflower genome. A total of 150 HaNACs were divided into 15 subgroups through phylogenetic analysis (Fig. 2), whereby genes performing the same function were clustered into the same group. For instance, the subgroups of *AtNAC3*, *NAP* and *ATAF* contained many classic stress-responsive *NAC* genes, including *ANAC019*, *ANAC056* (*AtNAC2*), *ANAC055* (*AtNAC3*), *ANAC002* (*ATAF1*), *ANAC081* (*ATAF2*), and *ANAC072* (*RD26*)^49,50^, suggesting that HaNACs in these three groups are likely to contribute to the stress response. Conserved motifs and gene structure also indicated that the same group of HaNACs might exert the same biological function (Fig. 3). These gene replication events are generally known to be crucial in the rapid expansion and evolution of gene families. Gene duplication of NAC TFs has been observed in many plant species. In our study, 27 pairs of gene replication events were generated in 150 *HaNACs*, which may significantly contribute to the expansion of the NAC family in sunflower (Table S3). The analysis of NTLs with transmembrane helixes in HaNACs was found to have similarity with the *Arabidopsis thaliana* NAC family members^51^, suggesting that they may function in specific locations by protease degradation via a similar mechanism as NTLs in *Arabidopsis*^52^ (Table 1, Figs. S3 and S4). Previous studies revealed that miRNA164s could direct mRNA cleavage of the *NAC* gene^53–55^. Our study showed that miR164 targeting sites might exist in 12 *HaNACs*, and miR164 possibly participates in the regulation of *HaNAC* gene expression.

*Cis*-elements play an important role in regulating gene expression^56,57^. In the promoter region of about 2000 bp upstream of *HaNAC* genes, many cis-acting elements were found, such as light-, methyl jasmonate-, anaerobic-, ABA-, and low temperature-responsive elements, indicating that *HaNACs* may be associated in sunflower photoperiod, stress and defense pathways. From the stress response perspective, the expression of *HaNAC* genes under PEG stress as revealed by RNA-seq data showed that some *HaNAC* genes had obvious expressions to drought stress (15% PEG stress). Through further analysis, we found that most of the stress-responsive NAC genes in the phylogenetic tree were clustered in the same subgroup, which was consistent with related studies in *Arabidopsis* and rice^58,59^. This indicated that *HaNAC* genes in the SNAC group may play a vital role in abiotic stress responses. Moreover, qRT-PCR results also showed that the expression of genes in the SNAC group significantly increased under NaCl and PEG stress, further confirming our previous results (SNAC genes are important in the abiotic stress response pathway). Besides, under the stress, *HaNAC70* and *HaNAC84* were more highly up-regulated in roots than leaves, whereas *HaNAC146* and *HaNAC105* were higher up-regulated in leaves. Additionally, *HaNAC55* showed a significant up-regulation in the Non-SNAC group, suggesting that the response of *HaNACs* to abiotic stress is complex or did not get enough research. Altogether, we can infer that sunflower *SNAC* genes are involved in the response of the plant to abiotic stress, and different SNAC genes may also play a role in different tissues.

## Conclusion

In this study, 150 sunflower *NAC* genes were identified, and their distribution, basic properties, classification, gene structure, evolutionary characteristics, and expression profiles were explored. Among them, eight potential *HaNTL* genes and 12 *HaNAC* genes that may be regulated by miR164s were found. Analysis of *cis*-acting elements and the expression profiles of *HaNACs* indicated that these genes were involved in response to salt and drought stress. A few important genes that may play an important role in tolerance of sunflower to abiotic stress have been speculated. Notably, this work can provide a solid basis for future functional studies of NAC to improve abiotic stress resistance in sunflower.

## Materials and methods

### Identification, physicochemical properties and chromosome distribution of sunflower NAC transcription factors

All protein sequences (version 1.0) of sunflower were downloaded from the EnsemblPlants database (http://plants.ensembl.org/). The HMM model file (PF02365) of the NAM domain in the pfam database (http://pfam.xfam.org/) was downloaded, and the software HMMER (version 3.2.1) was used to search for NAC proteins from all protein sequences of sunflower. The obtained proteins were verified in the CCD database (https://www.ncbi.nlm.nih.gov/cdd/). The remaining protein sequences with the complete NAC domain were aligned using the ClustalW method in MEGA X.

The Sequence Manipulation Suite (http://www.detaibio.com/sms2/) was used to analyze the basic physical and chemical properties of HaNACs, such as amino acid number, isoelectric point, and molecular weight.

The position of these genes on the chromosome was determined from the gtf file (version 1.0.42) in the Ensembl Plants database (http://plants.ensembl.org/). MapGene2Chrom web service (http://mg2c.iask.in/mg2c_v2.0/) was used for drawing chromosome distribution, and the pie chart was generated using Excel.

### Membrane-bound HaNACs and miR164 target site prediction

The CELLO website (version 2.5) was used to predict the subcellular localization of HaNACs, whereas the transmembrane helix of HaNACs was predicted with TMHMM 2.0 (http://www.cbs.dtu.dk/services/TMHMM-2.0/).

Sunflower miRNA164s and HaNACs mRNA sequences were input to psRNATarget (http://plantgrn.noble.org/psRNATarget/) to predict the action site of miRNA164. The picture was drawn using the gene structure display service (GSDS2.0 http://gsds.cbi.pku.edu.cn/).

### Phylogenetic, genetic structure and conserved motifs

The NAC protein sequences of sunflower and *Arabidopsis* were compared by ClustalW in MEGA X, and a phylogenetic tree was constructed using the Neighbour-Joining method of MEGA X. Then, the reliability of the tree was tested using 1,000 bootstrap tests.

The structures of the *HaNAC*s could be found in the gtf file. The MEME local software (version 5.0.4) of the motif analysis tool was used to find conservative motifs among members of the sunflower NAC family. Except for the maximum number of motifs which is 15, the other parameters were default. TBtools was applied to merge and draw evolution trees, genetic structures, and motifs^60^.

### Syntenic and evolutionary analysis and *cis*-element analysis

The syntenic relationship of *HaNAC*s was analyzed by the Multiple Collinearity Scan Toolkit (MCScanX). Here, potential gene pairs (E value < 10^–5^, first five matches) were obtained in the genome of sunflower through BLASTP as input files to analyze segmented and tandem duplications. After that, the relationship map between *HaNACs* was drawn using TBTools. DnaSP (version 6.12.03) was used to calculate the non-synonymous rate (Ka) and synonymous rate (Ks) of the homologous genes. The type of selection was determined according to the ratio of Ka and Ks, whereas the time of duplication events (T) was determined according to the following formula: T = Ks/2λ, where λ = 1.5 × 10^−8^ for dicots^61^.

Further, 2000 bp upstream of the start codon of the *HaNAC* gene was extracted as an input file for PlantCare (http://bioinformatics.psb.ugent.be/webtools/plantcare/html/), then *cis*-acting elements were analyzed, and we used TBTools to generate the picture.

### RNA-seq data download and expression analysis

Sunflower RNA-seq data for the expression analysis of different tissues and PEG stress were derived from SRA data (Bioproject: PRJNA483306; PRJNA398727). The expression was calculated, and the DEGs were analyzed according to the protocols described by Pertea et al.^62^ on the software including Hisat2 (version 2.1.0), Stringtie (version 1.3.5) and Ballgown (version 2.18.0). The heat map was generated using TBTools software.

### Plant materials and stress treatment

The sunflower used in this research was named as ZADT (Zaoaidatou), the backbone parent for breeding in Xinjiang, China. To understand the expression pattern of *HaNAC* genes under abiotic stress, seeds were sown, and seedlings were grown in a chamber at the photoperiod of 16 h/day 26 ℃ and 8 h/night at 20 ℃ for 16 days. Then, the seedlings with 4 true leaves were subjected to salt- (150 mM NaCl) and drought- (15% PEG6000) stressed treatments for 24 h, here the control was designed by water treatment. Sunflower roots and leaves were collected and frozen in liquid nitrogen for RNA extraction to detect the expression pattern of *HaNAC* genes under abiotic stress.

### RNA extraction and qRT-PCR

Total RNA was isolated using RNAprep pure Plant Kit (Tiangen, DP432). Reverse transcription was carried out using the PrimeScript™ 1st Strand cDNA Synthesis Kit (Takara, 6110A). The primers (listed in Table S7) were designed using the NCBI Primer-BLAST tool (https://www.ncbi.nlm.nih.gov/tools/primer-blast/), and *actin* was used as the internal reference gene. Power SYBR™ Green PCR Master Mix (Applied Biosystems, 4367659) and Applied Biosystems QuantStudio 5 Real-Time PCR Systems were used for qRT-PCR. The 2^−ΔΔCT^ method was used to calculate the expression level. Each qRT-PCR procedure was conducted with three biological replicates.

## Supplementary Information

Supplementary Figures.

Supplementary Tables.

## Data Availability

Our study complies with relevant institutional, national, and international guidelines and legislation. Permissions were obtained from Xinjiang Academy of Agricultural Sciences for collecting and using sunflower plants for this study. All plant materials used in this study were provided by Xinjiang Academy of Agricultural Sciences. RNA-seq fastq files were deposited in NCBI Sequence Read Archive (SRA) under accession number PRJNA483306 and PRJNA398727. Genome, proteome, and annotation files were available in EnsemblPlants database.
