# Supplementary material for: Genome-wide identification and comprehensive analysis of the NAC transcription factor family in sunflower during salt and drought stress
Source: Sci Rep. 2021 Oct 6;11:19865. doi: 10.1038/s41598-021-98107-4 (PMC8494813; doi:10.1038/s41598-021-98107-4)
Supplement: Supplementary file 1 — Supplementary Figures. [file 41598_2021_98107_MOESM1_ESM.pdf]

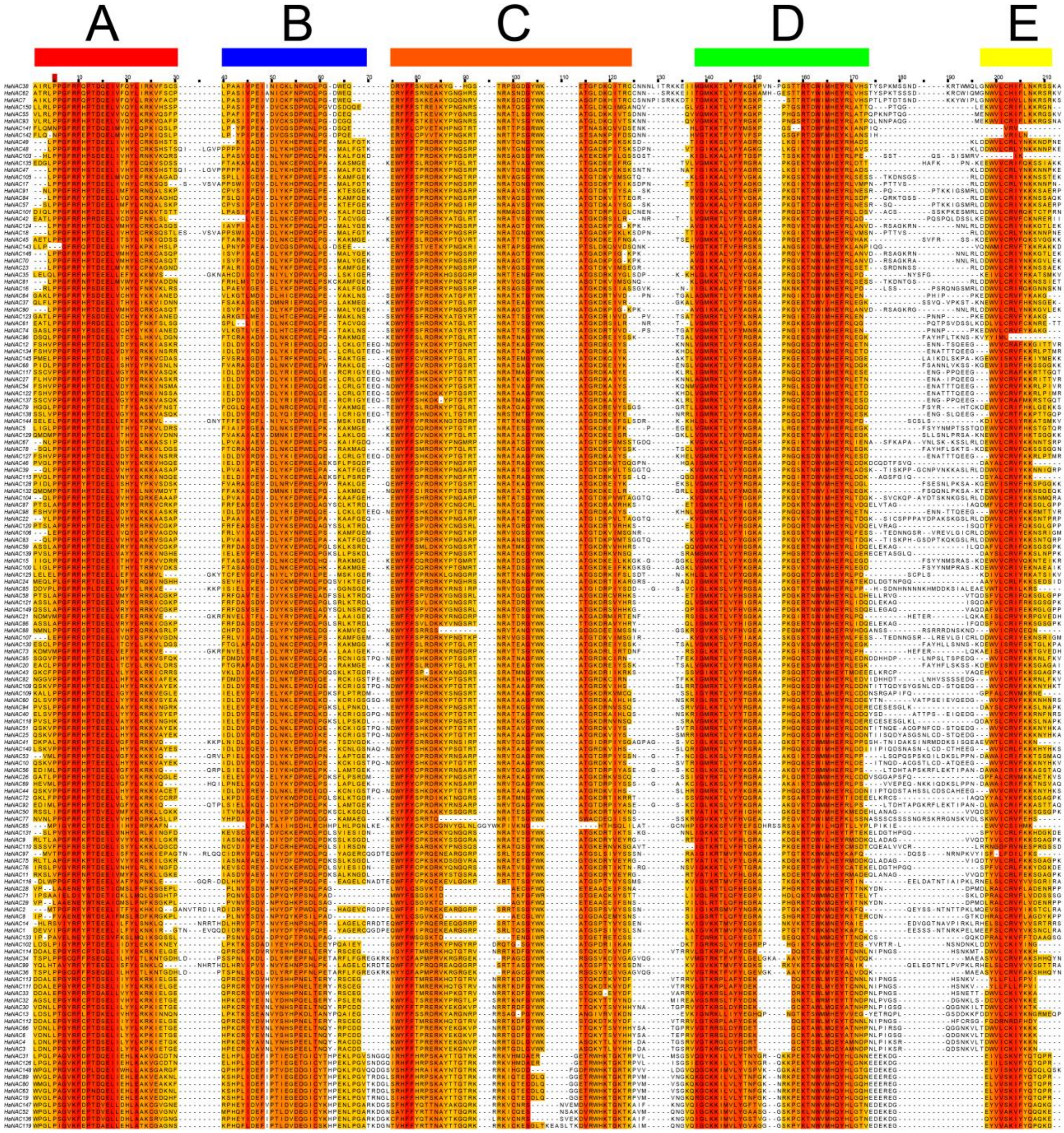

Fig. S1. Five subdomains and multiple sequence alignments of sunflower HaNAC transcription factors.

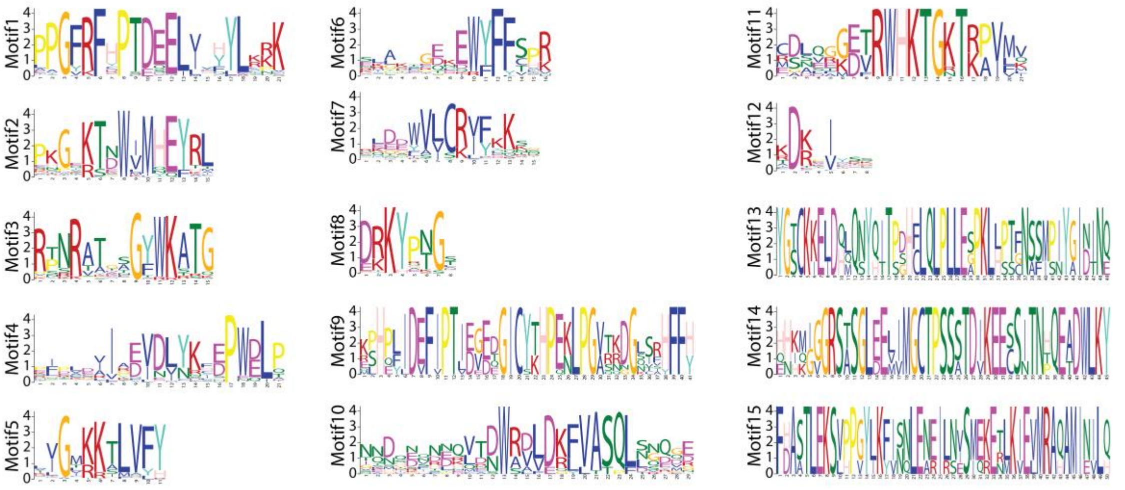

Fig. S2. ~~15~~ The motifs of HaNAC transcription factors using meme software.

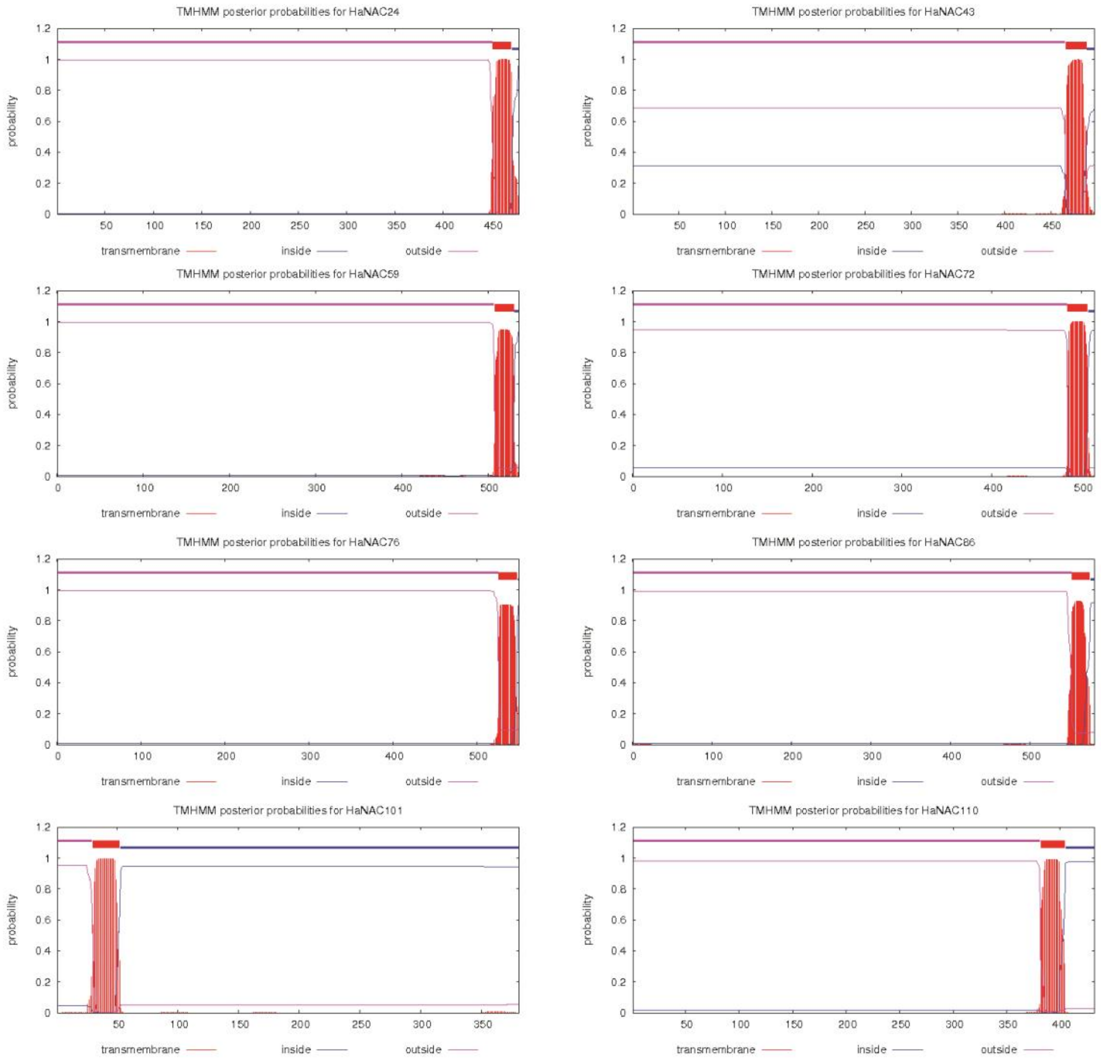

Fig. S3. Position of the transmembrane helix in eight HaNTLs.

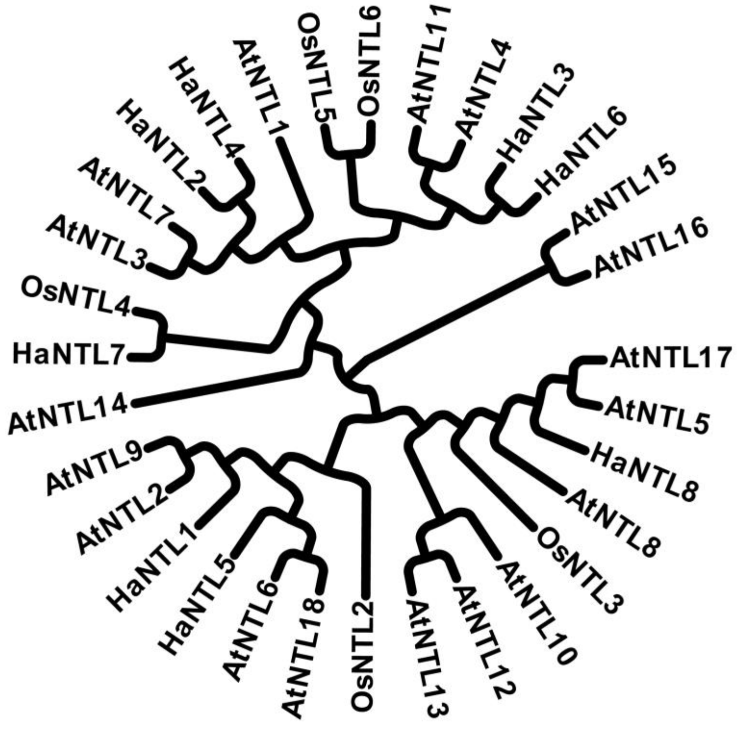

Fig. S4. Phylogenetic trees of NTLs in sunflower, *Arabidopsis* and rice.

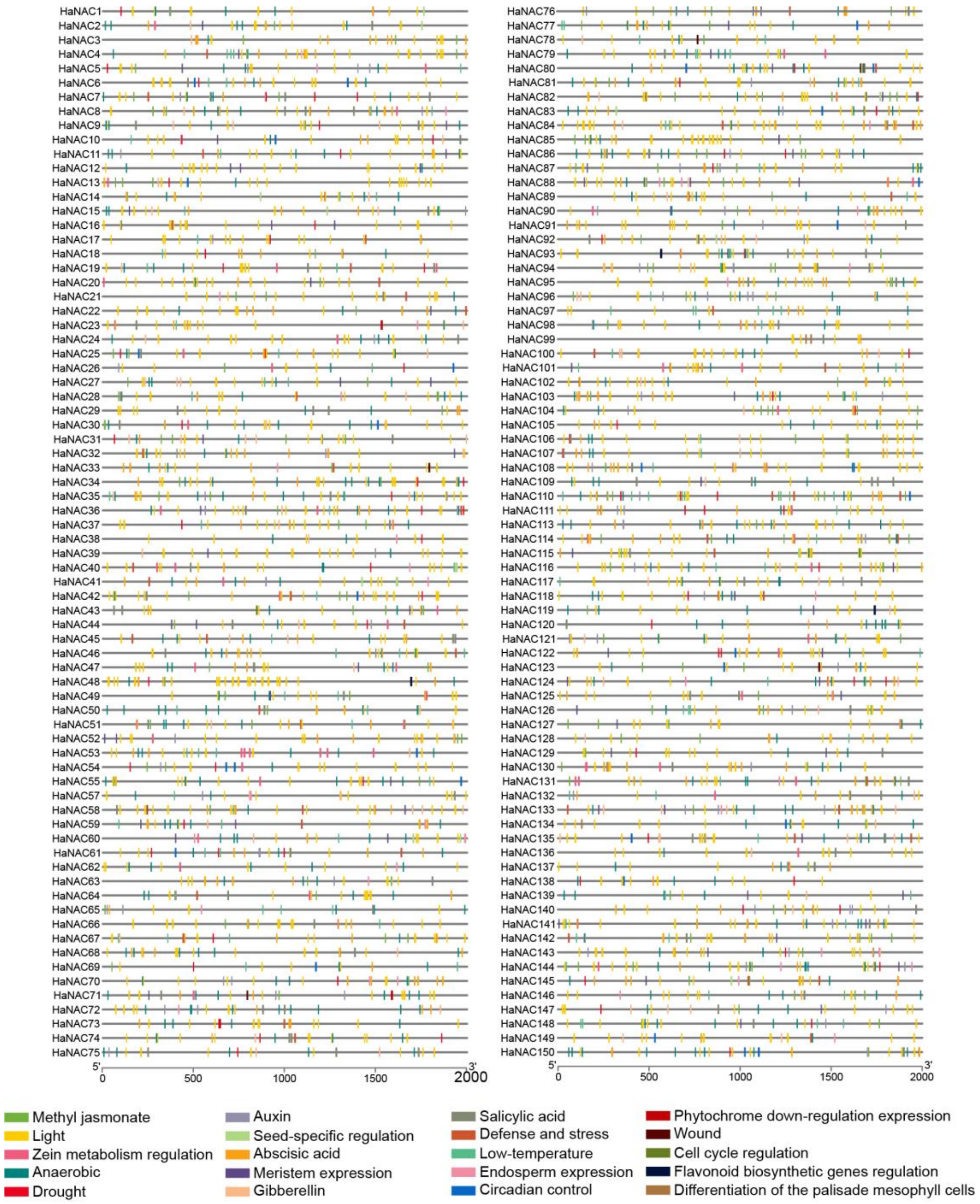

Fig. S5. *Cis*-elements in the promoter of *HaNAC* genes (2,000 bp upstream of CDS).
